# Complete Genome Sequence of *Lysinibacillus sphaericus* B1-CDA, a Bacterium That Accumulates Arsenic

**DOI:** 10.1128/genomeA.00999-15

**Published:** 2016-01-21

**Authors:** Aminur Rahman, Noor Nahar, Jana Jass, Björn Olsson, Abul Mandal

**Affiliations:** a Systems Biology Research Center, School of Bioscience, University of Skövde, Skövde, Sweden; b The Life Science Center, School of Science and Technology, Örebro University, Örebro, Sweden

## Abstract

Here, we report the genomic sequence and genetic composition of an arsenic-resistant bacterium, *Lysinibacillus sphaericus* B1-CDA. Assembly of the sequencing reads revealed that the genome size is ~4.5 Mb, encompassing ~80% of the chromosomal DNA.

## GENOME ANNOUNCEMENT

The arsenic-resistant strain B1-CDA was isolated from arsenic-contaminated land in Bangladesh (1). Sequencing of the genomic DNA of B1-CDA was performed by an Illumina HiSeq 2500 PE100 sequencer with a single sequencing index. The genome assembly started with Illumina 100-bp paired-end reads of genomic DNA with an insert length of 300 bp. The read quality was checked using FastQC (2). The raw reads were quality trimmed and corrected using Quake (3). Properly paired reads ≥30 bp in length were selected from the pool of corrected reads, and the remaining singleton reads were considered single-end reads. Both types of reads were then used in *k*-mer-based *de novo* assembly by employing SOAP*denovo* (4). The set of scaffolds with the largest *N*_50_ was identified by evaluating *k*-mers ranging from 29 to 99. The optimal scaffold sequences were further subjected to gap closing by utilizing the corrected paired-end reads. The resulting scaffolds of length ≥300 bp were chosen as the final assembly (5).

A total of 11,105,899 pairs of reads were generated by Illumina deep sequencing. Analysis of the raw reads with FastQC showed that the average per base Phred score was ≥32 for all positions, and the mean per sequence Phred score was 38. The overall G+C content was 38%. After quality trimming, error correction, and removal of the TruSeq adapter sequence, 10,940,654 read pairs (98.5%) and 145,888 single-end sequences remained for further analysis. The set of scaffold sequences with maximal *N*_50_ (507,225 bp) was produced at a *k*-mer of 91. The corresponding scaffold sequences were subjected to gap closure using the corrected paired-end reads, and the resulting scaffolds (≥300 bp) were defined as the final assembly. The final assembly was 4,509,276 bp, and it consisted of 31 scaffolds ranging from 314 bp to 1,145,744 bp.

The assembled genome sequence was annotated with RAST (6). The RAST analysis pipeline uses tRNAscan-SE to predict tRNA genes (7) and the Glimmer algorithm to predict protein-coding genes (8). Predictions of tRNA-, rRNA-, and protein-coding genes were performed based on 77 RAST-predicted tRNA genes. RAST resulted in 11 rRNA genes, including seven 5S, one 16S, and three 23S genes. A total of 4,513 protein-coding genes were predicted using the Glimmer algorithm, of which 2,671 protein-coding genes were annotated by RAST’s automated homology analysis and assigned to functional categories. GeneMark (9) and FgenesB (10) algorithms were also applied, yielding 4,562 and 4,323 genes, respectively. The functional annotation by RAST and Blast2GO (11) indicated that B1-CDA contains many genes, which are responsive to metal ions, like arsenic, cobalt, copper, iron, nickel, potassium, manganese, and zinc. All protein-coding sequences resulting from GeneMark were used by Blast2GO for functional annotation. Based on the phylogenetic trees inferred by using the neighbor-joining method (12) presented in the MEGA6 software (13), B1-CDA resembles *Lysinibacillus sphaericus* G10, R-27024, and CICR-X12.

In summary, strain B1-CDA demonstrates the presence of several metal-responsive genes that might be utilized in bioremediation of toxic metals in polluted environments.

### Nucleotide sequence accession numbers.

The genome sequence of B1-CDA strain has been deposited in GenBank under the accession number LJYY00000000. The version described in this paper is the first version, LJYY00000000.1.
